# Social relations and contact with general practitioner in a middle-aged Danish population: a prospective register- and survey-based cohort study

**DOI:** 10.1186/s12913-022-07658-3

**Published:** 2022-04-11

**Authors:** Anne Sophie Bech Mikkelsen, Rikke Lund, Volkert Siersma, Terese Sara Høj Jørgensen, Ulla Christensen, Maria Kristiansen

**Affiliations:** 1 Department of Public Health & Center for Healthy Aging, Section of Health Services Research, University of Copenhagen, Copenhagen, Denmark; 2 Department of Public Health & Center for Healthy Aging, Section of Social Medicine, University of Copenhagen, Copenhagen, Denmark; 3 Department of Public Health, The Research Unit for General Practice and Section of General Practice, University of Copenhagen, Copenhagen, Denmark; 4 Department of Public Health, University of Copenhagen, Section of Social Medicine, Copenhagen, Denmark

## Abstract

**Background:**

Findings about the relationship between individuals’ social relations and general practitioner (GP) contact are ambiguous as to whether weak social relations are associated with an increased or decreased consultation pattern. Furthermore, social relations may affect GP contact differently for men compared to women, between socioeconomic groups and according to perceived need. The overall aim of the study is to examine the association between functional aspects of social relations, perceived emotional and instrumental social support, the tendency to consult a GP and the frequency of GP contact.

**Methods:**

The study comprised 6911 individuals aged 49–61 at baseline from the Copenhagen Aging and Midlife Biobank (CAMB). We conducted a two-part regression to explore the association between perceived emotional and instrumental social support and GP contact (tendency and frequency), controlling for age, sex, occupational social class, cohabitation status and number of morbidities.

**Results:**

Results show no overall effect of the perceived social support aspects of social relations on GP contact independent of health-related needs.

**Conclusions:**

Our results do not support that perceived social support, reflecting functional aspects of social relations, are associated with general practitioner contact among middle-aged people.

**Trial registration:**

The study has been registered and approved by the Danish Data Protection Agency and the local ethical committee (approval No.H-A-2008-126 and No. 2013-41-1814).Keywords: social relations, perceived social support, healthcare utilisation, general practitioner, middle-aged

## Background

The social life of human beings is complex as reflected in decades of research within social epidemiology . Throughout the years, many different terms have been used to capture aspects of social life, including social networks, social relations, social support, social isolation and social integration [1]. Attempts have been made to define and clarify the different terms, for instance by Due et al. [2] who describes social relations as a neutral common term, covering structural and functional aspects such as emotional and instrumental social support [2]. Across different conceptualisations of social relations, there seems to be an agreement on defining social relations or social networks by the structure (quantity) and function (quality) of an individual’s social life. Structural aspects may be those of network size, diversity and contact frequency, whereas the functional aspects may be those related to the provision of emotional and instrumental support. Hence, structural aspects may reflect the extent to which a person is integrated into a social network, whereas the functional aspects reflect specific supportive functions in a person’s relationships, for example emotional or instrumental social support [1–6]. In this paper, social relations are viewed as a concept, which may affect healthcare utilisation and it is operationalised through measures of emotional and instrumental social support.

Researchers have argued that social relations and their different aspects such as emotional and instrumental social support shape a range of physical and psychological health measures, as well as overall morbidity and mortality throughout adult life. For example people with weak or strained social relations have been found to have higher morbidity and mortality than people with stronger social relations [7]. However, we know much less about how social relations relate to healthcare utilisation. There seems to be inadequate research and hence, ambiguous findings on the association between social relations and primary healthcare use such as contact to general practitioner (GP) [8, 9]. A recent systematic review found that studies on how social relations affect GP contact show conflicting findings as to the direction of the relationship [9]. One stream of research supports the hypothesis that weak social relations lead to an increased consultation pattern: a hypothesised mechanism is that people with weak social relations also have lower levels of self-reported health, which may cause them to contact their GP more often than people with stronger social relations [10, 11]. Moreover, it has also been argued that contact with a GP might be a way of fulfilling unmet social needs related to loneliness or social isolation [12]. A contrasting hypothesis is that adults with weak social relations might in fact use less healthcare because they lack aspects of social support such as financial help with transport, or emotional support that are barriers for timely access to healthcare services [3, 9]. The Danish healthcare system provides universal, publicly financed healthcare with no out of pocket payments except from dental care and a few other services. Primary care facilities run by GPs are the first point of contact for residents, and GPs act as gatekeepers as they may refer patients to e.g. specialist or hospitals. Each resident is registered at a specific GP practice, which they are free to choose and hence, the common pattern is, that individuals see the same GP for years and only re-register with a different GP when moving to a different municipality, if the GP retire, or if the individual is not satisfied with the treatment received. The social support aspects of social relations may play a larger role in an individual’s decision-making in regard to contacting the GP than in the decision-making regarding more specialised healthcare which – as in the Danish healthcare system – may need a referral. Behavioural, psychological and biological pathways are suggested to explain how social support – as an aspect of social relations - affects health outcomes. For instance, social support has been found to affect health behaviour and practices such as physical activity, smoking, alcohol consumption, sleep quality and compliance with treatment [4]. Psychological pathways include stress appraisal, depression and quality of life and can act as mediating factors while at the same time being significant health outcomes in themselves [4]. While most evidence on the relationship between social support and physical health relates to the mediating role of behavioural factors, there is also some evidence of a direct link between social support and health-related biological processes [4].

Differences according to sex have been found in a study showing that close relationships defined as being ‘strong attachment’ as well as ‘social and emotional support’ are more beneficial for men than for women [13]. Also, men with large social networks have been found to be more likely to contact their GP, relative to women with large social networks [9, 14]. Unsurprisingly, health status has been found to be the strongest predictor of primary healthcare use [14, 15]. This finding may be supported by the stress-buffering model, which states that social relationships primarily affect health-related outcomes among people who experience stressful events such as illness; and that the perception that one’s relations will provide support when needed is important [3]. Furthermore, only a few studies have investigated the possible differing effect of social support on GP contact according to measures of socioeconomic position, showing inconsistent results. One study found that education was unrelated to frequency of GP contact [15], whereas other studies have found income and education to have an effect on frequency of visits to physicians [16, 17]. Generally, the lack of unified findings leads to some unanswered questions as to the nature of the relationship between social relations and GP contact, the underlying mechanisms, and possible differential effects [8, 9]. Altogether, this points to the relevance of investigating whether social relations affect GP contact in different ways: for men and for women; for groups of varying health status; and – as a measure of socioeconomic position in this particular population – for various groups of occupational social classes.

In this study, we operationalise social relations as described in the conceptual framework by Due et al. and we focus specifically on the emotional and instrumental aspects of social support and how these constructs might affect healthcare utilisation [2]. We apply measures of perceived emotional and instrumental social support and investigate the association with tendency and frequency of GP contact. This way, we seek to explore how perceived emotional and instrumental social support affects the tendency to consult a GP, and the frequency of GP contact in a middle-aged Danish population (aged 49–61 at baseline).

Furthermore, we investigate whether there is differential vulnerability regarding perceived emotional and instrumental social support, according to sex, socioeconomic position, and according to health status. In the present study, we hypothesise that middle-aged individuals with low emotional and instrumental social support tend to contact their GP more, relative to their counterparts with high emotional and instrumental social support. Also, we hypothesise that this association might be more pronounced for specific sub-groups: in particular men relative to women; for individuals with at least one morbidity relative to persons with no morbidities; and among those in low socioeconomic positions relative to individuals in high socioeconomic positions.

## Methods

### Data sources

**Fig. 1:**
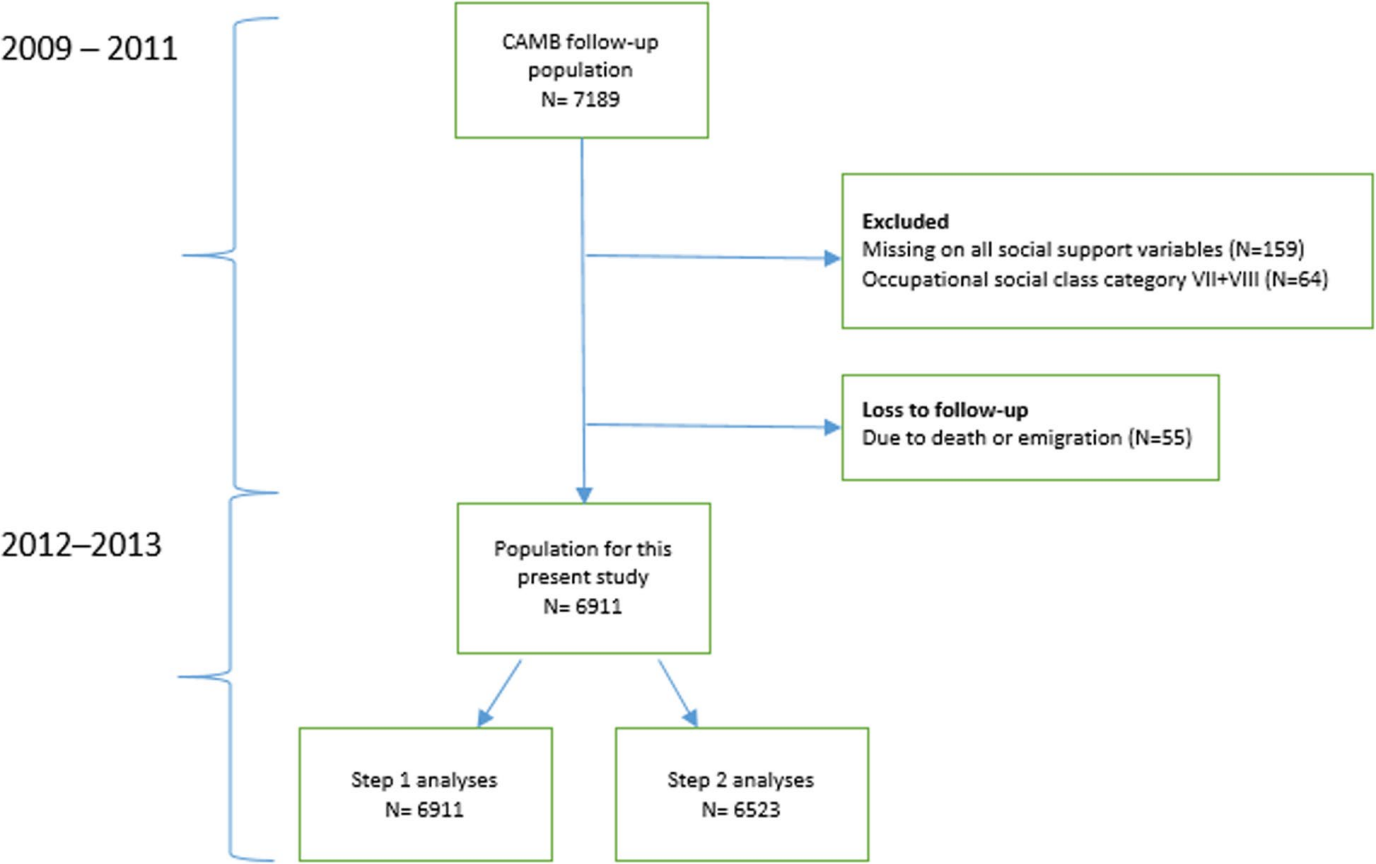
Flowchart of the selection of the study population

We linked questionnaire data from the Copenhagen Aging and Midlife Biobank (CAMB), collected in 2009–2011, with data on GP contacts in 2012–2013, from Danish administrative registries. The registries are part of an administrative system containing information about the activities of health professionals contracted within the tax-funded public primary healthcare system, for example, GPs [18, 19]. Within a short delimited period, such as the follow-up period chosen in this study, we assume perceived social support to be relatively stable for this particular group of adults, which is why the measures of perceived social support are hypothesized to affect GP contact during follow-up. The CAMB cohort consists of participants from earlier established cohorts; the Metropolit 1953 Danish Male Birth Cohort (MP), the Copenhagen Perinatal Cohort (CPC) born 1952–61, and the Danish Longitudinal Study on Work, Unemployment and Health (DALWUH) born 1949 or 1959. Figure 1 illustrates the process of defining the study population for the present analyses. From the eligible population of questionnaire respondents (N=7189) for this study we excluded 55 respondents due to death and emigration before start of follow-up, 159 respondents due to missing data and 64 respondents due to the exclusion of occupational social class category 7 and 8. Finally, 6911 respondents were included in the present analyses (Figure 1).

### Outcome

There were two primary outcomes in the analyses; GP contact (binary yes/no variable) and the total number of GP contacts (count variable) in the two-year follow-up period 2012–2013. Types of contacts included in the GP contact variable were physical consultations, telephone consultations, email consultations and patient home visits during regular hours. As the GP contact variable was derived from the Danish National Health Service Registry (DNHSR), it includes information on contacts such as date and type of consultation but no information on disease or conditions of the patient contacting the GP [18]. We derived variables from the registry on historical migrations (VNDS), and from the registry on all deaths in Denmark (DOD), to compute an offset variable enabling us to account for individuals who had either died or migrated during the follow-up.

### Variables

The exposures of interest were the functional aspects of social relations, operationalised as two variables measured at the CAMB questionnaire: perceived emotional social support and perceived instrumental social support. Perceived emotional social support was measured by the question: ‘Can you talk with any of the following people if you need support?’ and perceived instrumental social support was measured by the question: ‘Would any of the following people help you with daily practical matters if necessary?’ For both emotional and instrumental social support, the same question was posed for three different social roles: partner, other family, and friends. For each of the questions the response categories were: always, often, sometimes, seldom, never, have none. We defined the social support variables separately for the two categories of perceived emotional and perceived instrumental social support, counting the number of social roles from whom social support was expected to be available ‘always’ or ‘often’ across the three social roles. Hence, the two perceived social support variables had values ranging 0–3, being the number of social roles from whom support was always or often expected to be available.

Several potential confounders were included. We retrieved sex and age from the general population registry (BEF), with age included as age at December 31st 2011. Number of morbidities as a measure of health status, occupational social class as a measure of socioeconomic position, and cohabitation status were included from the CAMB questionnaire. Number of morbidities was defined as the sum of the presence of the following 21 conditions: asthma, allergy, diabetes, cataract, hypertension, myocardial infarction or angina pectoris, stroke, chronic bronchitis, osteoarthritis, osteoporosis, peptic ulcer, cancer, migraine or frequent headaches, chronic anxiety or depression, other mental disorder/bad nerves, back disease, urinary incontinence, difficulty urinating, tinnitus, kidney stones and gallstones. Socioeconomic position was measured by the Danish Occupational Social Class Measure (DOSCM), which is a measure of socioeconomic position applicable in late mid-life populations, computed from self-reported information on income level and occupation. The measure is based on assessments of the occupational skills and competencies necessary for the job as well as the power and control associated with the position [20]. This measure consists of eight categories: social class I (highest): jobs which require top-level educational attainment (at least 4 years of university or similar training); social class II: white-collar jobs that require approximately 3 years of theoretical training (e.g. nurse, primary school teacher, journalist etc.); social class III: non-manual white-collar jobs which demands expertise at basic level and self-employed at small scale businesses;social class IV: manual white-collar jobs which require some theoretical training up to 1 year as well as practical training; social class V: manual jobs which require little theoretical and practical training including semi- or unskilled workers; social class VI: people who are economically inactive and rely primarily on transfer income (e.g. disability pensioner, unemployed or long term sick); social class VII: a special category which include people who are economically active but with insufficient information to classify them according to the Social Class Classification; and social class VIII: a special category including students andstay-at-home partners [20]. For the present analyses we excluded category VII, due to insufficient information to place in a social class group, and we excluded category VIII, based on an assumption that this group was too different from the others in terms of social relations (N=158). We collapsed the remaining six categories into four: social classes I–II, social classes III–IV, social class V and social class VI. We did this, as the job types classifying the Social Class Classification are similar in terms of level of theoretical and/or practical training for social class I and II and for social class III and IV, respectively. Social class V and VI primarily are too different – unskilled workers versus people on transfer income - and hence, did not make sense to collapse in the present analyses. . Cohabitation was measured by the question: ‘Do you live alone?’.

### Statistical analyses

The analysis of the association between social relations and GP contact was conducted in a two-part model for the purpose of separating the healthcare-seeking behaviour of the patient from the healthcare contacts allocated by healthcare professionals. First, we fitted a model that analysed the tendency to make contact with the GP, i.e. extrapolating this from the number of contacts with a GP, and second, we fitted a model that analysed the number of contacts for those who have any GP contact [21]. The part one model used a modified Poisson regression to estimate the association of social relations with the tendency to contact a GP, possibly adjusted for certain potential confounders, as a set of incidence ratios (IRs) compared to the baseline social support category [22]. The part two model used a generalised linear regression model, assuming gamma distributed residuals and a logarithmic link function, to analyse the association between social relations and the number of GP contacts, in those that had at least one GP contact. The effect measures in this model were rate ratios (RRs) of how much more the service was used compared to the baseline social support category. To adjust for differing follow-up times, in both the part one and part two models the logarithm of the length of time the patient was observed in the two years 2012–2013 (i.e. living in Denmark and not dead) was included as offset. The IR from the part one model and the RR from the part two model were multiplied to get a combined effect indicating how many more GP contacts were observed in one social support category compared to the reference social support category [21, 23–25].

We conducted three analyses; first, a crude model of the association between social support and GP contact, only adjusted for person years; second, a model where we additionally adjusted for age, sex, cohabitation status, and occupational social class; and third, a model where we also adjusted for number of morbidities. These three analytical models were set up in order to investigate the effect of the confounding variables. As health status is the strongest predictor of healthcare utilisation, we wished to investigate the effect of number of morbidities separately from the other confounding variables.

Interactions were tested in both model parts separately to investigate whether the effect of social support differed between groups according to sex, comorbidity and occupational social class. For the purpose of the interaction analyses, we dichotomised the measures of social relations into 0–1 vs. 2–3 social roles perceived to provide social support always or often.

Individuals with missing values for one or more variable were omitted from the analyses where these variables were included. Statistical significance was tested at a 5% level. We used SASv9.4 for analyses.

## Results

Table 1 shows that the majority had at least one source of close social relation perceived to provide support (always/often). For emotional social support there were relatively more people who had three sources of close social relations perceived to provide support (42.2 %), than for instrumental social support (27.9 %). For instrumental social support, having one source of close social relation providing support accounted for the largest proportion (35.4 %). It appears from this that having three sources of close social relations perceived to provide support (always/often) served as a suitable reference category. Moreover, few individuals had no contacts at all with their GP in the follow-up period 2012–2013 (5.6 %) and the distribution was relatively even across all co-variables.

**Table 1:** Baseline characteristics and outcome at follow-up *N* (%)

	Total	Tendency to contact GP *N* =6911		Number of GP contacts among those with a minimum of one contact *N*= 6523
		No	Yes	Mean (SD)
Total	6911	388 (5.6)	6523 (94.4)	11.9 (11.9)
Emotional social support^a^				
0	472 (6.8)	24 (5.1)	448 (94.1)	13.8 (13.4)
1	1635 (23.7)	95 (5.8)	1540 (94.2)	12.7 (13.5)
2	1887 (27.3)	119 (6.3)	1768 (93.7)	11.7 (11.7)
3	2917 (42.2)	150 (5.1)	2767 (94.9)	11.4 (10.7)
Instrumental social support^a^				
0	865 (12.5)	46 (5.3)	819 (94.7)	14.0 (14.5)
1	2444 (35.4)	134 (5.5)	2309 (94.5)	11.7 (11.5)
2	1674 (24.2)	107 (6.4)	1567 (93.6)	11.7 (12.1)
3	1928 (27.9)	100 (5.2)	1828 (94.8)	11.4 (10.7)
Age				
50–57	2898 (41.9)	123 (4.2)	2775 (95.8)	11.8 (12.1)
58–63	4013 (58.1)	264 (6.6)	3748 (93.4)	12.0 (11.7)
Sex				
Males	4780 (69.2)	348 (7.3)	4431 (92.7)	11.0 (11.2)
Females	2131 (30.8)	39 (1.8)	2092 (98.2)	14.0 (12.9)
Copenhagen Occupational Social Class				
I–II	2683 (38.8)	174 (6.5)	2509 (93.5)	9.6 (9.1)
III + IV	2696 (39.0)	137 (5.1)	2558 (94.2)	11.3 (10.0)
V	680 (9.9)	44 (6.5)	636 (93.3)	12.9 (11.7)
VI	784 (11.3)	27 (3.4)	757 (96.6)	20.4 (19.5)
Missing	68 (1.0)	5	63	
Cohabitation (Do you live alone?)				
Yes	1145 (16.6)	76 (6.6)	1069 (93.4)	13.8 (14.7)
No	5732 (82.9)	308 (5.4)	5423 (94.6)	11.5 (11.2)
Missing	34 (0.5)	3	31	
Comorbidity/No. of diseases				
0	2189 (31.7)	197 (9.0)	1991 (91.0)	8.4 (7.9)
1	2093 (30.3)	118 (5.6)	1975 (94.4)	10.4 (9.9)
2	1351 (19.5)	50 (3.7)	1301 (96.3)	12.7 (11.6)
3+	1278 (18.5)	22 (1.7)	1256 (98.3)	19.1 (16.3)

^a^Number of sources of social relations that always/often provide emotional and instrumental support in case of need

### Main analyses

In Table 2, we present incidence ratios (IR), rate ratios (RR) and combined effects of the association between perceived emotional and instrumental social support and GP contact. Overall, estimates were close to the reference value— having three sources of social relations perceived to provide support always or often— and none were statistical significant (p<0.05) when adjusting for age, sex, occupational status and number of morbidities. This indicate the variables included as confounders and modifying variables accounted for differences observed in the first model (Table 2) in GP contacts, according to levels of perceived emotional and instrumental social support.

**Table 2:**
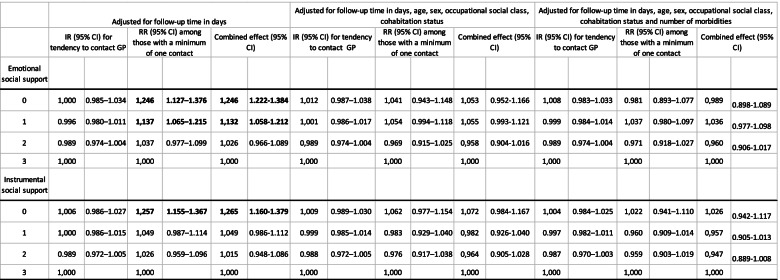
Incidence rates (IR) and rate ratios (RR) with 95% confidence intervals (95% CI) for the association between social relations and contact with GP

Bold values indicates statistically significant *p*–values (*p*<0.05)

### Interaction analyses

In Table 3, we present incidence ratios (IR) and rate ratios (RR) of interaction parameters of each of the two main exposure variables and the co-variables sex, number of morbidities and occupational social class. In the interaction analyses, we dichotomised the measures of social relations into 0–1 vs. 2–3 social roles perceived to provide social support always or often. Except for the analyses of the effect of perceived instrumental social support according to men and women, groups of occupational social class, and groups with varying number of morbidities, and the analyses of the effect of perceived emotional social support according to groups of occupational social class. However, most of the estimates were insignificant and all were close to the reference category. Statistically significant estimates were found for those who had 1 morbidity where low perceived emotional social support was associated with a lower incidence rate of having any GP contact at all (0.967, 95% CI 0.946–0.988), relative to having 1 morbidity and high perceived emotional social support; for those who had 3+ morbidities, where low perceived instrumental social support was associated with a lower rate ratio of GP contact (0.992, 95% CI 0.984-0.999), compared to GP contacts of those who had 3+ morbidities and high perceived instrumental social support; and for occupational social class VI, where low perceived instrumental social support was associated with a lower rate ratio of GP contacts ( 0.979, 95% CI 0.964–0.994), compared to the GP contacts of those in occupational social class VI, with high perceived instrumental social support. Together with the overall insignificant results from the main analyses, I argue that the interaction analyses show no strong indications of differential effects of perceived emotional and instrumental social support.

**Table 3:** Incidence rates (IR) and rate ratios (RR) with 95% confidence intervals (CI) for effect measure modifications

	Emotional social support						Instrumental social support					
	IR (95% CI) for tendency to contact GP		RR (95% CI) among those with a minimum of one contact		Combined effect (95% CI)		IR (95% CI) for tendency to contact GP		RR (95% CI) among those with a minimum of one contact		Combined effect (95% CI)	
	Adjusted IR (95% CI)^a^		Adjusted RR (95% CI)^a^				Adjusted IR (95% CI)^a^		Adjusted RR (95% CI)^a^			
Sex												
Males	0.993	0.976–1.011	0.997	0.992–1.001	0.990	0.972-1.008	**0.989**	**0.973–1.005**	**0.996**	**0.992–1.000**	0.985	0.969-1.001
Females	0.998	0.984–1.011	1.002	0.997–1.006	1.000	0.987-1.014	**1.009**	**0.996–1.022**	**1.002**	**0.998–1.006**	1.011	0.998-1.024
Number of morbidities												
0	**1.027**	**0.994–1.062**	0.999	0.993–1.005	1.026	0.991-1.061	1.005	0.979–1.033	**0.999**	**0.995–1.004**	1.004	0.976-1.032
1	**0.967**	**0.946–0.988**	0.998	0.994–1.004	0.965	0.944-0.987	0.992	0.971–1.013	**1.003**	**0.999–1.007**	0.995	0.974-1.016
2	**0.999**	**0.974–1.024**	0.999	0.990–1.009	0.998	0.972-1.025	0.986	0.964–1.008	**0.994**	**0.985–1.003**	0.980	0.957-1.004
3+	**0.986**	**0.968–1.005**	0.994	0.984–1.003	0.980	0.960-1.000	0.996	0.979–1.013	**0.992**	**0.984–0.999**	0.988	0.970-1.006
Occupational social class												
Social class I–II	0.999	0.980–1.019	1.002	0.998–1.007	1.001	0.981-1.022	0.994	0.976–1.012	**1.001**	**0.997–1.004**	0.995	0.977-1.013
Social class III–IV	1.038	0.991–1.088	1.000	0.983–1.008	1.038	0.990-1.089	1.035	0.994–1.078	**1.003**	**0.999–1.007**	1.038	0.997-1.081
Social class V	1.000	0.932–1.073	1.000	0.963–1.038	1.000	0.923-1.083	1.000	0.958–1.020	**0.991**	**0.981–1.001**	0.991	0.969-1.013
Social class VI	0.996	0.974–1.018	1.002	0.998–1.005	0.998	0.976-1.020	0.993	0.974–1.013	**0.979**	**0.964–0.994**	0.972	0.948-0.997

^a^Adjusted for all variables in the full model i.e. sex, age, number of morbidities, occupational social class and cohabitation status

Bold values indicate statistically significant *p*-values for interaction terms (*p*<0.05)

## Discussion

We investigated two main questions in this prospective cohort study, conducted in a middle-aged Danish population (aged 49–61 at baseline). First, we investigated how perceived emotional and perceived instrumental social support is associated with GP contact tendency, and the numbers of GP contacts and secondly, whether there is differential vulnerability according to sex, number of morbidities, and according to occupational social class; that is, whether there is differential vulnerability. Overall, we found that perceived emotional and instrumental social support dimensions of social relations were not significantly associated with GP contact in this adult population and that there were no strong indications of differential vulnerability.

To our knowledge, there are no previous studies specifically measuring the perceived emotional and instrumental dimensions of functional aspects of social relations in relation to GP utilisation, and generally, we found few studies investigating how functional aspects of social relations are associated with GP utilisation. In the following, we will therefore relate our findings to those from studies applying other functional measures of social relations, such as social integration and social anchorage among middle-aged and older people [8, 14–17]. Two previous studies on social integration and social anchorage found no association with GP utilisation in individuals aged 50 years and older, nor in individuals aged 60–78 years, respectively [15, 26]. On the other hand, the latter study also found that a sense of community cohesion and belonging was associated with a higher frequency of GP use. However, with only a small effect, leading the authors to conclude that social- and psychological factors only influence GP use marginally; and that comorbidity was the strongest predictor of frequent GP use [15]. Moreover, in contrast with our findings that instrumental social support is not associated with GP contact, a study found that receiving and providing financial support increased both the likelihood and number of GP visits, among people of 60 years and older. However, in the same study, emotional social support was not associated with GP utilisation [17]. In line with results published by Bremer et al. 2018, we find no strong indications of interactions between emotional and instrumental social support and comorbidity. Bremer et al. did not find any interaction effect between comorbidity and the functional aspect of social integration on GP contact. However, they did find differential effects of social contact frequency and number of emotionally close relationships according to health status (measured as self-rated health). Among people with good health status, high social contact frequency was associated with more GP visits. Finally, among people with poor health, a higher number of emotionally close relationships were associated with more GP visits [26]. Moreover, our findings are in line with those of Korten et al. who did not find that social support was associated with the volume of GP contacts for either men or women. Moreover, they found that the strongest predictor for healthcare utilisation was physical health [14]. On the other hand, Korten et al. find indications of low (relative to high) social support being associated with a lower tendency to contact a GP among men compared to women [14].

### Strength and limitations

The large study population, and the prospective nature of the study design, are two major strengths of this study, giving substantial weight to the results and diminishing the risk of reverse causality [27]. Another strength of this study is the application of detailed register data of high validity to measure contacts with GPs. Applying these objective measures of GP contacts throughout the two-year study period eliminates the risk of recall bias as opposed to if a questionnaire based measure of GP contacts had been applied [18].

The measures of social support included in the analyses have been validated for content and face validity as well as for reliability, and it has been argued that they are suitable for measuring functional aspects of social relations, specifically among middle-aged individuals [5]. To our knowledge, there is no validated measure combining the emotional and instrumental social support measures, and therefore we believe it is appropriate to conduct separate analyses for the two types of social support respectively. Moreover, the social support aspects of social relations may play a larger role in an individual’s decision-making in regard to contacting the GP than in the decision-making regarding more specialised healthcare that – as in the Danish healthcare system – may need a referral. Hence, GP contact is a reasonable outcome measure when studying how social support affects healthcare utilisation. A further strength is the inclusion of the Danish Occupational Social Class Measure (DOSCM) as a measure of socioeconomic position. The DOSCM is based on an assessment of the occupational skills and competencies necessary for a particular job, and the influence and control associated with the position. The measure has been demonstrated to be suitable specifically to the late middle-aged, as this group might be transitioning from working age to old age. Moreover, the use of DOSCM – rather than separate measures of income, education and occupation – enables analysis of possible effects of material resources, combined with skills and knowledge linked to social standing in society [20].

The study also has, nevertheless, potential limitations that warrant attention. Although the study population is relatively large, we cannot completely rule out that the insignificant findings were a result of lack of statistical power. Moreover, as described elsewhere, the CAMB population is a selective population. Compared to non-participants, a larger proportion of participants were employed, male, and had Danish origin. Furthermore, only people living in a specific geographic area (defined as ‘the eastern parts of Denmark’) were invited to participate [28, 29]. Moreover, non-respondents showed a higher all-cause mortality than respondents throughout the follow-up [28, 29]. Yet, participants did not differ substantially from non-participants, either regarding number of contacts with GP or educational level, during the first year of follow-up [28, 29]. We conducted a sensitivity analysis including the 278 individuals excluded due to missing values in the full population, in which the results did not differ substantially. In the analyses, it was not possible to account for possible changes in level of emotional and instrumental social support during the two-year follow-up period. To limit the bias possibly arising from this and which might diminish or erase an existing association, it might have been appropriate to choose a shorter follow-up, namely one year or six months. Moreover, we included a self-reported measure of health status: number of (self-reported) morbidities. This might have biased the results, particularly if the effect of health on GP contact is either stronger or weaker than measured through these self-reported measures, or if individuals either forget or leave out health information when filling out the questionnaire. However, as we find no reasons to assume that this possible measurement error relates to the perceived emotional social support or perceived instrumental social support, it is unlikely that it has had any particular effect on the results. In relation to this, an ideal alternative to the self-reported measure of health status would have been objectively registered diagnoses and conditions related to each of the registered GP contacts. However, as the DNHSR is established mainly for administrative purposes it only contains information on type of consultation (physical, telephone, email etc.) and no information on the reason for contact. It is possible that the pattern found in this study is somewhat blurred by not knowing whether the contacts reflect routine consultations or conditions requiring more intense medical treatment. If for instance, the majority of health conditions in this population sample were of a serious nature requiring intense treatment, it might be that the seriousness of the condition, rather than the perceived level of social support, were the main driver of GP contact. In the Danish primary care system, each citizen is registered to a single dedicated general practice. As such, it is rare that a person contacts another general practitioner or visits different practices. Hence, for practical purposes in a statistical analysis, persons are nested within practices. It is likely that some practice characteristics may make their patients consult their GP more/less. Without specifying these characteristics, heterogeneity between practices with respect to these will make observations within practices dependent. This could have been adjusted for in the analysis employing the GEE approach covering whole practices, not just the individual patients (as implemented now). Such additional adjustment would not affect the point estimates, only their standard errors and thereby the width of their confidence intervals. In the current study, we did not implemented these adjustments. This is primarily because we did not have access to these data and gaining access would have involved considerable time and administrative efforts. Moreover, using a proxy for practice, e.g. municipality, would render an adjustment with little effect. Furthermore, the dependences between patients within practices are unlikely to be strong: predictors for health care seeking behavior are typically characteristics of the patient rather than the practice, e.g. cohabitation, geographical distance, and there are guidelines in place to regulate and standardize follow-up for specific groups of patients. Therefore, we argue that the dependence within practices is not substantial. Another potential limitation is the question of novelty as our survey data were collected in 2009-2011, and the follow-up data on GP contacts refer to the period 2012-2013. However, as the healthcare system in Denmark has not changed markedly in terms of how it is organized and structured during the last ten years (and more), we do believe that the data applied in this paper are still relevant. Finally, but still very important to consider, is the age of the sample, which is 49-61 years at baseline. Middle-aged adults may be much less dependent on others to seek healthcare than for example older adults and altogether this might be part of the reason why we found no associations between perceived emotional and instrumental social support and GP contact. In sum, it would indeed be interesting to replicate the analyses in a sample of older people and including objective measures of health status.

### Implications and future research

Generally, our findings do not support the argument that perceived social support as a functional aspect of social relations, independent from health-related needs, are associated with general practitioner contact among middle-aged people. Hence, this study does not give weight to the promotion of social interventions to strengthen social relations and social support, reducing social isolation and loneliness, among middle-aged people with low social support. However, in the future it would be valuable to replicate the study with several GP follow-up points, and to include more of the functional aspects of social relations such as relational strain and social anchorage. In this way, it might be possible to explore how changes in social support over time affect contact with GPs, and how different functional aspects might be a stronger or lesser predictor of GP contact over time. Furthermore, it would be valuable to conduct in-depth qualitative studies to better understand the mechanisms between perceived social support and GP contact among middle-aged people.

## Conclusion

Our findings show that perceived instrumental and emotional social support are not associated with general practitioner contact – neither the tendency to make contact nor the number of contacts. The effect sizes of the associations are relatively close to the reference value, and when adjusting for possible confounding and modifying factors all of the investigated associations are statistically insignificant. Moreover, investigation of two-way interactions with sex, number of morbidities, and socioeconomic position, showed no strong indications for differential vulnerabilities.

## Data Availability

Restrictions, set out by the Danish Data Protection Agency, apply to the availability of data, which we used under license for the study, and so are not publicly available. Throughout the project period, data was securely stored at a remote researcher server at Statistics Denmark [30], which is a state organisation administering and regulating access to statistical and administrative registries in Denmark. Accessing the project data located at the remote server, for analysis in this paper, relied on several security steps.

## Abbreviations

GP: General practitioner
CAMB: Copenhagen Aging and Midlife Biobank
MP: Danish Male Cohort
CPC: Copenhagen Perinatal Cohort
DALWUH: Danish Longitudinal Study on Work, Unemployment and Health
DNHSR: Danish National Health Services Register
VNDS: Register on historical migrations and health
DOD: Register on all deaths in Denmark
BEF: General population register
DOSCM: Danish Occupational Social Class Measure
IR: Incidence rates
RR: Rate ratios

## References

[1] Berkman LF, Kawachi I, Glymour MM, Berkman LF, Krishna A (2014). Social Network Epidemiology. I: Social Epidemiology.

[2] Due P, Holstein B, Lund R, Modvig J, Avlund K (1999). Social relations: network, support and relational strain. Soc Sci Med.

[3] Cohen S (2004). Social Relationships and Health. Am Psychol.

[4] Uchino BN, Bowen K, de Grey RK, Jude M, Fischer EB (2018). Social Support and Physical Health: Models, Mechanisms and Opportunities. I: Principles and Concepts of Behavioral Medicine.

[5] Lund R, Nielsen LS, Henriksen PW, Schmidt L, Avlund K, Christensen U (2014). Content Validity and Reliability of the Copenhagen Social Relations Questionnaire. J Aging Health.

[6] Lund R, Christensen U, Iversen L (2011). Medicinsk sociologi: sociale faktorers betydning for befolkningens helbred.

[7] Holt-Lunstad J, Smith TB, Layton JB (2010). Social Relationships and Mortality Risk: A Meta-analytic Review. PLoS Medicine.

[8] Bremer D, Inhestern L, von dem Knesebeck O (2017). Social relationships and physician utilization among older adults-A systematic review. PLoS One.

[9] Valtorta NK, Moore DC, Barron L, Stow D, Hanratty B (2018). Older Adults’ Social Relationships and Health Care Utilization: A Systematic Review. Am J Public Health.

[10] Cornwell EY, Waite LJ (2009). Social Disconnectedness, Perceived Isolation, and Health among Older Adults. J Health Soc Behav.

[11] Xu F, Johnston JM (2015). Self-Rated Health and Health Service Utilization: A Systematic Review. Int J Epidemiol.

[12] Ellaway A, Wood S, Macintyre S (1999). Someone to talk to? The role of loneliness as a factor in the frequency of GP consultations. Br J Gen Pract.

[13] Orth-Gomér K (2009). Are social relations less health protective in women than in men? Social relations, gender, and cardiovascular health. J Soc Person Relation.

[14] Korten AE, Jacomb PA, Jiao Z, Christensen H, Jorm AF, Henderson AS (1998). m.fl. Predictors of GP service use: a community survey of an elderly Australian sample. Australian New Zealand J Public Health.

[15] Rennemark M, Holst G, Fagerstrom C, Halling A (2009). Factors related to frequent usage of the primary healthcare services in old age: findings from The Swedish National Study on Aging and Care. Health Soc Care Commun.

[16] Arling G (1985). Interaction Effects in a Multivariate Model of Physician Visits by Older People. Med Care.

[17] Li Y, Chi I (2011). Correlates of Physician Visits Among Older Adults in China: The Effects of Family Support. J Aging Health.

[18] Andersen JS, Olivarius NDF, Krasnik A (2011). The Danish National Health Service Register. Scand J Public Health.

[19] Pedersen CB (2011). The Danish Civil Registration System. Scand J Public Health.

[20] Christensen U, Krølner R, Nilsson CJ, Lyngbye PW, Hougaard CØ, Nygaard E (2014). m.fl. Addressing Social Inequality in Aging by the Danish Occupational Social Class Measurement. J Aging Health.

[21] Diehr P, Yanez D, Ash A, Hornbrook M, Lin DY (1999). Methods for Analyzing Health Care Utilization and Costs. Ann Rev Public Health.

[22] Zou G (2004). A Modified Poisson Regression Approach to Prospective Studies with Binary Data. Am J Epidemiol.

[23] Jørgensen TSH, Lund R, Siersma VD, Nilsson CJ (2017). Interplay between financial assets and social relations on decline in physical function and mortality among older people. Eur J Ageing.

[24] Rasmussen JF, Siersma V, Pedersen JH, Heleno B, Saghir Z, Brodersen J (2014). Healthcare costs in the Danish randomised controlled lung cancer CT-screening trial: A registry study. Lung Cancer.

[25] Waldorff FB, Siersma V, Waldemar G (2009). Association between subjective memory complaints and health care utilisation: a three-year follow up. BMC Geriatr.

[26] Bremer D, Lüdecke D, Vonneilich N, von dem Knesebeck O (2018). Social relationships and GP use of middle-aged and older adults in Europe: a moderator analysis. BMJ Open.

[27] Rothman KJ (2012). Epidemiology, an introduction.

[28] Avlund K, Osler M, Mortensen EL, Christensen U, Bruunsgaard H, Holm-Pedersen P (2014). Copenhagen Aging and Midlife Biobank (CAMB) An Introduction. J Aging Health.

[29] Lund R, Mortensen EL, Christensen U, Bruunsgaard H, Holm-Pedersen P, Fiehn N-E. Cohort Profile: The Copenhagen Aging and Midlife Biobank (CAMB). Int J Epidemiol. 2015:dyv149. 10.1093/ije/dyv149 26210613

[30] Data for research [Internet]. [henvist 9. juli 2020]. Tilgængelig hos: https://www.dst.dk/en/TilSalg/Forskningsservice

[31] Datatilsynet [Internet]. [henvist 30. juni 2020]. Tilgængelig hos: http://www.datatilsynet.dk/

